# Lobelia—Ism—Its Prospects and Policy

**Published:** 1860

**Authors:** J. Dickson Smith

**Affiliations:** Macon, Ga.


"*Lobelia—Ism—its Prospects and Policy.*—By J. Dickson Smith, M. D., of Macon, Ga.

We have received a pamphlet of 16 pages bearing the above title, which is intended to show that the authors of the few works which have appeared from the leaders of Thompsonianism have stolen all their material, except the treatment, and in some instances even that, from the books of the regular profession. It is specially complimentary to what is called the "Reform Medical Practice," published by the Reform Medical College of Macon, Ga., as, it is stated, the *only* Thompsonian College now in existence. This book is said to contain extensive and undeniable plagiarisms, purloined from Watson, Wood and Dunglison, the very language of these authors being copied to the extent of twenty pages consecutively, without the slightest reference to the source from which the matter was obtained. This and a great deal more stated in the pamphlet of Dr. J. Dickson Smith, is doubtless true, and we heartily wish him all possible success in his efforts to show up the rascality of these wholesale plunderers, but for ourselves, we would about as soon be engaged in following thieves into cess-pools and sewers, in search of stolen treasures.

